# A supervised machine learning approach to predicting subjective cognitive decline (SCD) among diverse Hispanics/Latinos: The SOL‐INCA Study

**DOI:** 10.1002/alz.091573

**Published:** 2025-01-09

**Authors:** Sayaka Kuwayama, Wassim Tarraf, Kevin A Gonzalez, Freddie Márquez, Frank J Penedo, Sylvia Wassertheil‐Smoller, Martha L Daviglus, Melissa Lamar, Linda C Gallo, Hector M González

**Affiliations:** ^1^ University of California, San Diego, La Jolla, CA USA; ^2^ Wayne State University, Detroit, MI USA; ^3^ University of Miami, Coral Gables, FL USA; ^4^ Albert Einstein College of Medicine, Bronx, NY USA; ^5^ University of Illinois at Chicago, Chicago, IL USA; ^6^ Rush Alzheimer’s Disease Center, Chicago, IL USA; ^7^ San Diego State University, San Diego, CA USA

## Abstract

**Background:**

Increasingly, research evidence is identifying subjective cognitive decline (SCD) as a precursor for cognitive impairment and dementia. Identifying predictors of SCD is essential for understanding its utility as a preclinical indicator for impairment and especially pertinent for Hispanics/Latinos who have limited access to healthcare resources and clinical diagnostics and are disproportionally affected by Alzheimer’s disease and related dementias. We extend work on predictors of Mild Cognitive Impairment (MCI) in diverse Hispanics/Latinos in the US by modeling multidomain predictors of SCD.

**Method:**

We use data (n = 4347, average baseline age = 56.4‐years) from the Hispanic Community Health Study/ Study of Latinos (HCHS/SOL; 2008‐2011; Visit 1), a multisite prospective cohort study of diverse Hispanics/Latinos, and its ancillary study, the SOL‐Investigation of Neurocognitive Aging (SOL‐INCA; average 7‐years after Visit 1). Our outcome is a composite SCD measured at SOL‐INCA by averaging the component items of the Everyday Cognition (ECog‐12) scale and is modeled using 37 cross‐domain Visit 1 indicators, previously linked to MCI, reflecting (1) sociodemographic characteristics, (2) childhood factors, (3) acculturation factors, (4) biological and (5) behavioral markers, and (6) mental and (7) functional health factors. We use supervised machine learning (ML: Random Forest = RF; regression = ML‐Reg) and standard statistical techniques (regression = Reg) for identifying leading predictors of SCD. In secondary analysis, we assess enhancement in predictive performance by accounting for Visit 1 global cognitive (GC) function.

**Result:**

Our best performing (i.e., R‐squared) ML model (ML‐Reg) explained only 17% of the variance in SCD. Leading identified predictors of SCD included physical health scores, airflow obstruction, anxiety, mental health scores, Hispanic/Latino heritage, education, depression, income, and language and social acculturation. GC was predictive of SCD and explained an additional 5% of the variance.

**Conclusion:**

Our findings indicate that multidomain factors contribute to SCD prediction, but the explained variance was relatively low. Biological markers, previously linked to MCI in our cohort, played a less significant role. Notably, the fit of the ML models for SCD was low relative to MCI specific models in the same population. Follow‐up work investigates how incorporating contemporaneous measures of the factors (vs. baseline alone) may improve the predictive capacity of the ML models.

